# Chemical pneumonitis and subsequent reactive airways dysfunction syndrome after a single exposure to a household product: a case report

**DOI:** 10.1186/1752-1947-3-112

**Published:** 2009-11-09

**Authors:** Imran Khalid, Amanda M Godfrey, Daniel R Ouellette

**Affiliations:** 1 Consultant, Department of Critical Care, King Faisal Specialist Hospital & Research Center, Jeddah, Saudi Arabia; 2 Division of Pulmonary and Critical Care Medicine, Henry Ford Hospital, W Grand Boulevard, Detroit, Michigan, 48202, USA

## Abstract

**Introduction:**

Household products are usually safe to use. Adverse events arising from their use are mostly reported in patients with pre-existing atopy or pulmonary problems and usually only after a prolonged exposure to such products. We report the case of a patient with no prior problems who developed significant side effects from a single exposure to a domestic product.

**Case presentation:**

A 43-year-old Caucasian American man, previously in good health, used a domestic aerosol product called 'Stand N' Seal "Spray-On" Grout Sealer' in an enclosed room in his house. The product contained n-butyl acetate (<5%), propane (10%), isobutane (<5%), C8-C9 petroleum hydrocarbon solvent (80%), a fluoropolymer resin and a solvent. Within a few hours of exposure to the sealant, he developed rapidly progressive shortness of breath and a severe non-productive cough. By the time he reached the emergency room he was severely hypoxic. A diagnosis of chemical pneumonitis was made based on the clinical scenario and the diffuse infiltrates on the computer tomography scan. With supportive therapy, his condition improved and he was discharged from the hospital. However, he continued to have symptoms of intermittent cough and shortness of breath in response to strong odours, fumes, cold air and exertion even after his chest radiograph had normalized. Three months later, bronchial hyper-responsiveness was documented by a methacholine inhalation test and a diagnosis of reactive airways dysfunction syndrome was made. The patient was started on high-dose inhaled steroids and his symptoms improved. The mechanism of toxicity and determination of the exact agent responsible is still under investigation.

**Conclusion:**

A household product may still prove unsafe to use even after it has gone through vigorous testing and approval processes. Even healthy individuals are susceptible to adverse outcomes after a brief exposure. Extra precautions should be taken when using any chemical product at home.

## Introduction

Household products are usually safe to use. Adverse respiratory events are most commonly experienced by patients with pre-existing atopy or pulmonary problems, and usually after a prolonged exposure to these products [1-5]. Cleaning agents, particularly bleach, are the most common culprits [2,3,5]. We report here, however, a case of a patient with no prior health problems who developed significant side effects from a single exposure to a domestic product, a grout sealer. To the best of our knowledge, this is the first case where a patient developed chemical pneumonitis and subsequent reactive airways dysfunction syndrome (RADS) from the combination of specific ingredients contained in this product.

## Case presentation

A 43-year-old Caucasian American man, who was a smoker but had no subjective or objective evidence of pulmonary disease or atopy, was in good health until he used a domestic aerosol product called 'Stand N' Seal "Spray-On" Grout Sealer' in an enclosed room in his house. Within a few hours of exposure to this sealant, he developed rapidly progressive shortness of breath and a severe non-productive cough. He did not have any associated chest pain or fever. His examination at our Emergency Department revealed a blood pressure of 155/89 mmHg, a heart rate of 126/minute, a respiratory rate 24/minute and a temperature of 37.2°C. No cyanosis, clubbing or edema was found. Lung auscultation revealed diminished air entry and inspiratory bilateral rales. A basic laboratory work-up showed normal results, including normal cell count differential.

**Figure 1:**
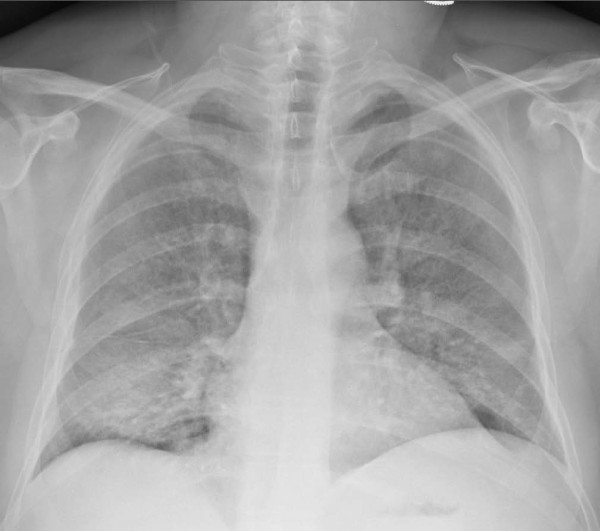
**Chest radiograph. Chest X-ray showing bilateral airspace disease**.

**Figure 2:**
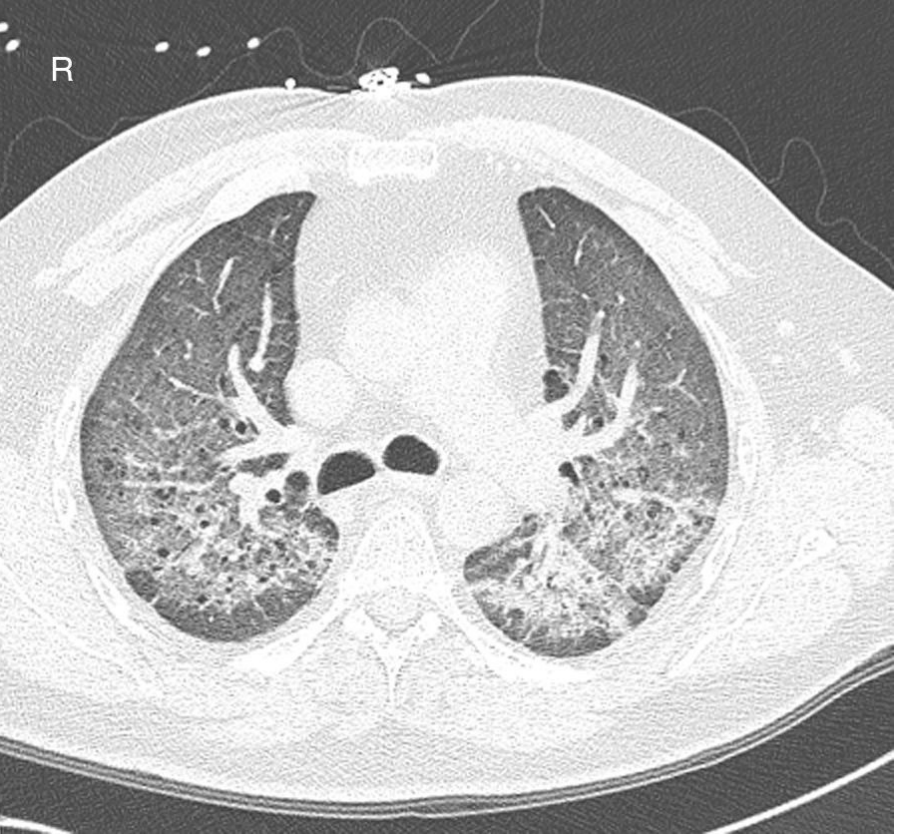
**Computed tomographic (CT) scan**. Non-contrast CT scan showing diffuse bilateral ground-glass opacities.

An electrocardiogram showed sinus tachycardia without any other abnormality. An arterial blood gas on room air demonstrated a PO_2 _of 32.7 mmHg and oxygen saturation of 67.2%, which improved to 85.2 mmHg and 97.4%, respectively, with 100% inspired oxygen. A chest X-ray and computer tomography of the patient were also obtained (Figures 1 and 2).

The patient was initially admitted to the intensive care unit to treat his chemical pneumonitis. He was initially treated with oxygen, albuterol nebulization and intravenous high-dose methylprednisolone, which was then followed by oral dexamethasone (10 mg every 8 hours). His condition improved in a few days. Upon discharge, he did not require oxygen. His respiratory symptoms had improved and he was instructed to take a 10-day course of oral prednisone.

The patient went to our pulmonary clinic one week after being discharged. He noted a definitive improvement in his symptoms but said that he was still experiencing intermittent wheezing and chest tightness, which could be alleviated by inhaling albuterol. He reported that he had not smoked tobacco since his hospitalization. A second chest X-ray yielded a normal result. A pulmonary function testing (PFT) showed reduced lung volumes and mild reduction in the patient's single-breath diffusion capacity for carbon monoxide (DLCO). He did have a significant bronchodilator response to albuterol (320 ml and 13% improvement in FEV1 (forced expiratory volume in 1 second)).

The patient returned for follow-up one month after presentation with complaints of an intermittent cough and shortness of breath in response to strong odours, fumes, cold air and exertion. He had not smoked tobacco since his hospital discharge. He then underwent cardiopulmonary exercise testing, which showed no ventilatory mechanical limitation, gas exchange abnormality or diffusion impairment. At about 3 months after his initial exposure to the cleaning product, the patient underwent a methacholine challenge test (MCT), which demonstrated a 22% decrease in his FEV_1 _level from 2.80 liters to 2.19 liters following the administration of methacholine at a concentration of 1 mg/ml. A diagnosis of reactive airways dysfunction syndrome (RADS) was thus made, as our patient fulfilled the seven diagnostic criteria for RADS [6].

**Figure 3:**
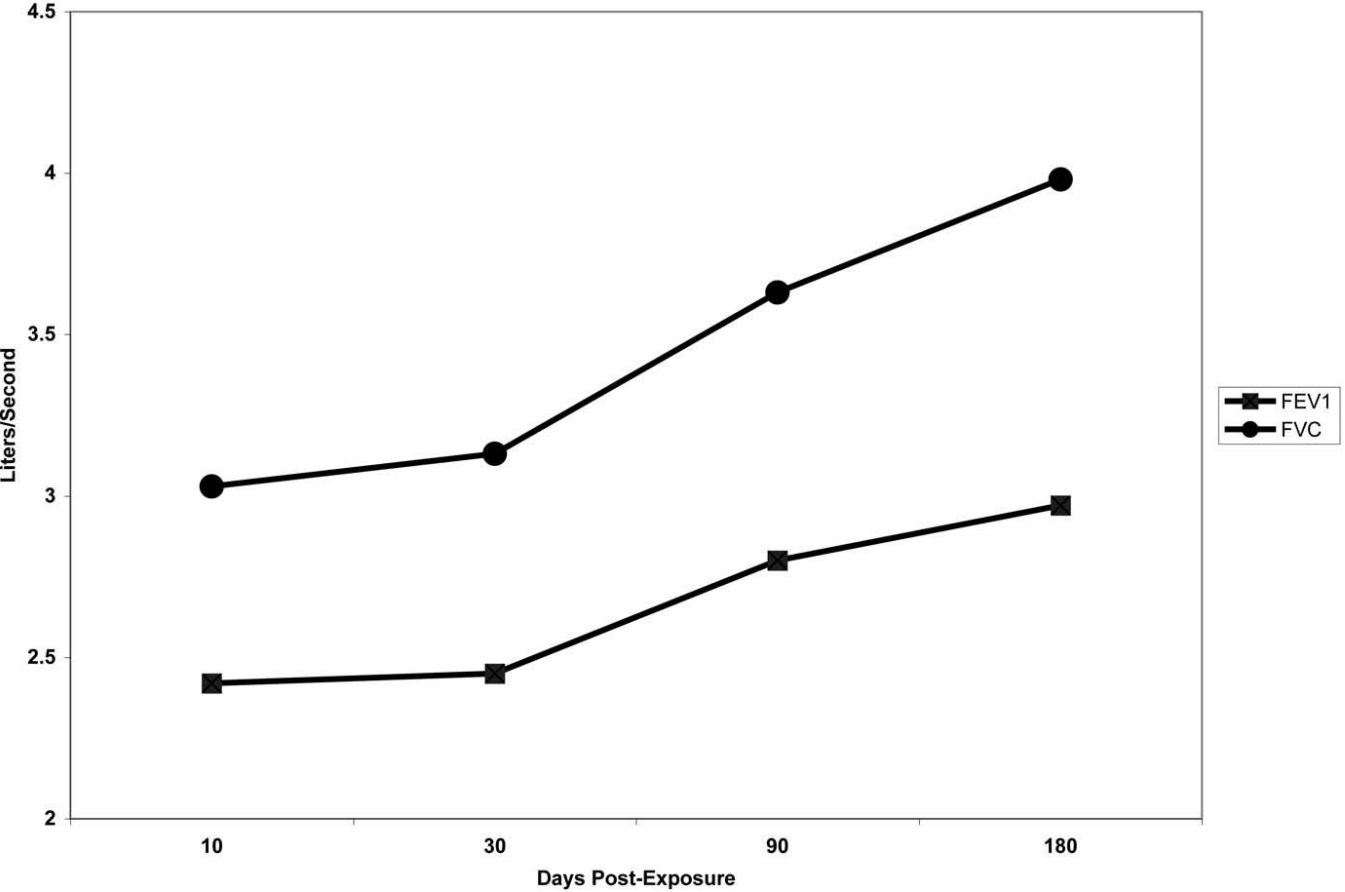
**Graph showing changes in pulmonary function testing with time after the initial exposure**. FEV1: forced expiratory volume in 1 second; FVC: forced vital capacity.

**Figure 4:**
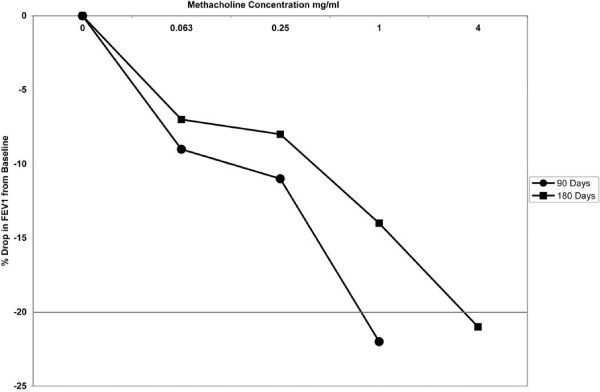
**Graph showing changes in methacholine challenge testing with time after the initial exposure**. FEV1: forced expiratory volume in 1 second.

The patient was started on inhaled fluticasone and salmeterol. His cough and shortness of breath in response to strong odours, fumes, cold air and exertion showed a slow but steady improvement. His PFT showed a gradual improvement in flows (Figure 3). Six months after the initial exposure to the chemical, the patient had a repeat MCT and his FEV_1 _decreased from 2.75 litres to 2.33 litres (21% reduction). However, the concentration of methacholine, which had to achieve a positive test, was now 4 mg/ml as opposed to the 1 mg/ml on the initial MCT (Figure 4). He was made to continue his therapy until his bronchial reactivity is resolved.

## Discussion

Household products are usually declared safe to use after they pass appropriate testing conducted by responsible agencies. Most of the studies on cases where patients have developed respiratory complaints after exposure to inhaled domestic products show that the conditions develop in patients with pre-existing lung problems or atopy [1-4]. Symptoms of asthma exacerbation, chronic bronchitis, bronchial hyper-responsiveness and acute respiratory distress syndrome have been reported in such patients [1-3]. One particular study shows that housewives can develop RADS after their continued exposure to bleach (40% sodium hypochlorite and 18% hydrochloric acid) [5]. However, the development of severe chemical pneumonitis and subsequent RADS in an individual who has no pre-existing lung condition and had been exposed only once to the component combination of this household product, has not been reported before.

Chemical pneumonitis has been reported after exposure to a variety of industrial chemicals and respiratory irritants. The treatment is usually supportive, although steroids have been administered during the acute phase after exposure. RADS is a nonimmunologic asthma-like syndrome resulting from a high level of exposure to an irritant gas, smoke, fume or vapour either at home, at the workplace or in the general environment [6,7]. The incidence of developing RADS after an inhalational exposure to an irritant substance has been difficult to quantify because patient-specific information on the magnitude and duration of exposure at the time of an inhalational accident is often not available.

The pathogenesis of RADS, particularly the persistence of the asthmatic state, is based on speculation. One hypothesis is that extensive inflammation associated with short-term exposure may alter receptor thresholds in the airways, thus resulting in non-specific bronchial hyperreactivity [7,8]. Other hypotheses involve direct damage to the bronchial mucosa and the release of mediators altering smooth muscle responsiveness, both eventually causing bronchial hyper-reactivity [7,8]. The pathologic features of RADS have also been difficult to define as data from serial bronchial biopsies starting at the time of exposure are limited. One case report describes serial histopathologic bronchial alterations of up to 5 months after chlorine inhalation, demonstrating that the histopathologic abnormalities are partially reversible [9].

Risk factors contributing to the development of RADS are also not well defined. In highly exposed rescue workers at the World Trade Center in New York, USA, bronchial hyper-reactivity at 1 and 3 months post-exposure was the sole significant predictor for the development of RADS [10]. In addition, increased concentrations of offending agents and wet aerosols are said to enhance the probability of developing RADS [11]. Management of patients with established RADS is based on minimal evidence. Many patients have been treated initially with oral corticosteroids, which were then followed by high-dose inhaled corticosteroids. Serial monitoring of bronchial hyper-reactivity is often advocated. Tapering of the inhaled corticosteroids is usually based on the clinical response to the treatment. The response to treatment is variable and the condition may take months or years to resolve [12].

The United States Consumer Product Safety Commission recalled 'Stand N' Seal "Spray-On" Grout Sealer' after receiving 88 reports of adverse reactions that developed following the use of this aerosolized sealant. The Commission report stated that the product's odour was not chemically pungent enough to force consumers to minimize their exposure to the fumes. A total of 28 individuals sought medical attention for respiratory symptoms before the product was recalled and 13 required medical treatment (Office of Information and Public Affairs. CPSC, Tile Perfect, Inc.: Announce Recall of Stand N' Seal Grout Sealer Due to Respiratory Problems. U.S. Consumer Product Safety Commission, 08/31/2005; Washington, DC.). 'Stand N' Seal "Spray-On" Grout Sealer' is composed of D-limonene, n-butyl acetate (<5%), propane (10%), isobutane (<5%) and C8-C9 petroleum hydrocarbon solvent (80%) (Tile Perfect, Inc. Stand N Seal Spray On Grout. Material Safety Data Sheet. 05/31/2005, Aurora, IL.). However, there appears to have been an alteration in the grout sealer's original composition when a different fluoropolymer and solvent were used, causing the allegation that the fluoropolymer resin may have actually caused the symptoms. The mechanism of toxicity and determination of the exact agent responsible, however, is still under investigation. The agents most frequently associated with the development of RADS include chlorine, toluene di-isocyanate and oxides of nitrogen [13]. Fluoropolymer inhalation can also cause acute pulmonary toxicity [14]. It is not evident which toxic substance led to the development of RADS in our patient, but fluoropolymers, isobutane and C8-C9 petroleum hydrocarbon solvent can all cause respiratory irritation [14,15].

## Conclusion

To the best of our knowledge, this may be the first official documentation of a patient developing chemical pneumonitis with subsequent RADS after a single exposure to the components of this specific sealer. The product lacked a pungent odour, which probably resulted in over-exposure to the vapours (Office of Information and Public Affairs. CPSC, Tile Perfect, Inc.: Announce Recall of Stand N' Seal Grout Sealer Due to Respiratory Problems. U.S. Consumer Product Safety Commission, 08/31/2005; Washington, DC.). However, whether it was a specific polymer in the product, a specific solvent or a mixture that caused the symptoms, needs to be further evaluated to avoid potential complications arising from the use of similar products. In the meantime, it is important to take precautions when using chemical products at home as household products may not be totally safe even after going through vigorous testing and approval processes.

## Abbreviations

DLCO: diffusion capacity for carbon monoxide; FEV1: forced expiratory volume in 1 second; PFT: pulmonary function testing; MCT: methacholine challenge test; RADS: reactive airways dysfunction syndrome.

## Consent

Written informed consent was obtained from the patient for publication of this case report and any accompanying images. A copy of the written consent is available for review by the Editor-in-Chief of this journal.

## Competing interests

The authors declare that they have no competing interests.

## Authors' contributions

IK wrote the case report portion of the manuscript. AMG wrote the discussion. DRO made critical revisions to the whole manuscript. All authors read and approved the final manuscript.

## References

[B1] Henderson J Sherriff A Farrow A Ayres JG Household chemicals, persistent wheezing and lung function: effect modification by atopy? Eur Respir J 2008 31 547 554 10.1183/09031936.00086807 17959633

[B2] Medina-Ramón M Zock JP Kogevinas M Sunyer J Torralba Y Borrell A Burgos F Antó JM Asthma, chronic bronchitis, and exposure to irritant agents in occupational domestic cleaning: a nested case-control study Occup Environ Med 2005 62 598 606 10.1136/oem.2004.017640 16109815 PMC1741089

[B3] Mapp CE Pozzato V Pavoni V Gritti G Severe asthma and ARDS triggered by acute short-term exposure to commonly used cleaning detergents Eur Respir J 2000 16 570 572 10.1034/j.1399-3003.2000.016003570.x 11028675

[B4] Anderson RC Anderson JH Respiratory toxicity of fabric softener emissions J Toxicol Environ Health A 2000 60 121 136 10.1080/009841000156538 10872633

[B5] Gorguner M Aslan S Inandi T Cakir Z Reactive airways dysfunction syndrome in housewives due to a bleach-hydrochloric acid mixture Inhal Toxicol 2004 16 87 91 10.1080/08958370490265004 15204781

[B6] Tarlo SM Balmes J Balkissoon R Beach J Beckett W Bernstein D Blanc PD Brooks SM Cowl CT Daroowalla F Harber P Lemiere C Liss GM Pacheco KA Redlich CA Rowe B Heitzer J Diagnosis and management of work-related asthma: American College Of Chest Physicians Consensus Statement Chest 2008 34 3 Suppl 1S 41S 10.1378/chest.08-0201 18779187

[B7] Brooks SM Weiss MA Bernstein IL Reactive airways dysfunction syndrome (RADS). Persistent asthma syndrome after high level irritant exposure Chest 1985 88 376 384 10.1378/chest.88.3.376 4028848

[B8] Alberts WM do Pico GA Reactive Airways Dysfunction Syndrome Chest 1996 109 1618 1626 10.1378/chest.109.6.1618 8769520

[B9] Lemiere C Malo J-L Boutet M Reactive airways dysfunction syndrome due to chlorine: sequential bronchial biopsies and functional assessment Eur Resp J 1997 10 241 244 10.1183/09031936.97.10010241 9032521

[B10] Banauch GI Alleyne D Sanchez R Olender K Cohen HW Weiden M Kelly KJ Prezant DJ Persistent hyperreactivity and reactive airway dysfunction in firefighters at the World Trade Center Am J Respir Cri Care Med 2003 168 54 62 10.1164/rccm.200211-1329OC 12615613

[B11] Kern DG Outbreak of the reactive airways dysfunction syndrome after a spill of glacial acetic acid Am Rev Respir Dis 1991 144 1058 1064 1952431 10.1164/ajrccm/144.5.1058

[B12] Malo JL Cartier A Boulet LP L'Archeveque J Saint-Denis F Bherer L Courteau JP Bronchial hyperresponsiveness can improve while spirometry plateaus two to three years after repeated exposure to chlorine causing respiratory symptoms Am J Respir Crit Care Med 1994 150 1142 7921449 10.1164/ajrccm.150.4.7921449

[B13] Shakeri MS Dick FD Ayres JG Which agents cause reactive airways dysfunction syndrome (RADS)? A systematic review Occup Med 2008 58 205 211 10.1093/occmed/kqn013 18308694

[B14] Lazor-Blanchet C Rusca S Vernez D Berry R Albrecht E Droz PO Boillat MA Acute pulmonary toxicity following occupational exposure to a floor stain protector in the building industry in Switzerland Int Arch Occup Environ Health 2004 77 244 248 10.1007/s00420-004-0505-6 15007653

[B15] Rohrig TP Sudden death due to butane inhalation Am J Forensic Med Pathol 1997 18 299 302 10.1097/00000433-199709000-00015 9290881

